# Prevalence and Causes of Functional Disability in an Elderly General Population of Japanese: The Hisayama Study

**DOI:** 10.2188/jea.JE20110083

**Published:** 2012-05-05

**Authors:** Daigo Yoshida, Toshiharu Ninomiya, Yasufumi Doi, Jun Hata, Masayo Fukuhara, Fumie Ikeda, Naoko Mukai, Yutaka Kiyohara

**Affiliations:** 1 Department of Environmental Medicine, Graduate School of Medical Sciences, Kyushu University, Fukuoka, Japan; 2 Department of Medicine and Clinical Science, Graduate School of Medical Sciences, Kyushu University, Fukuoka, Japan

**Keywords:** functional disability, dementia, stroke, prevalence, Japanese elderly

## Abstract

**Background:**

There are limited data on the prevalence and causes of disability in the elderly general population in Japan.

**Methods:**

In a population-based cross-sectional study of 1550 Japanese aged 65 years or older, we examined the prevalence of functional disability (defined as a Barthel Index score of ≤95) and its causes.

**Results:**

A total of 311 of the participants had a disability (prevalence 20.1%). The prevalence of disability increased with age and doubled with every 5-year increment in age. Prevalence was higher in women than in men, especially among those aged 85 years or older. With respect to the cause of functional disability, dementia accounted for 23.5%, stroke for 24.7%, orthopedic disease for 12.9%, and other disease for 38.9% of cases in men; in women, the respective values were 35.8%, 9.3%, 31.0%, and 23.9%. Regarding age, dementia was the most frequent cause of disability in subjects aged 75 years or older, whereas stroke was most common in subjects aged 65 to 74 years. Approximately two-thirds of cases of total dependence were attributed to dementia in both sexes, whereas the main cause of slight or moderate/severe dependence was stroke in men and orthopedic disease in women. Among participants with total dependence, 94.8% resided in a hospital or health care facility.

**Conclusions:**

Our findings indicate that functional disability is common among Japanese elderly adults and that its major cause is stroke in men and dementia in women.

## INTRODUCTION

The elderly population has been rapidly increasing worldwide, especially in developed countries. In Japan, the proportion of adults aged 65 years or older among the whole population has been the highest in the world since 2004, and it reached 23.0% in 2010.^1^ Along with this aging population, an increase in functional disability, which causes dependency and institutionalization, is a serious social, medical, and economic concern.^2^^,^^3^ Studies of the prevalence, causes, and effects of functional disability among the elderly population are therefore needed for appropriate public health policy and planning. Several community-based studies have reported the prevalence of functional disability and its causes in the elderly in Western countries^4^^–^^9^ and Japan.^10^^–^^14^ However, participants staying in hospitals or health care facilities were not surveyed in those studies, which likely led to underestimation of the prevalence of disability. Furthermore, information from questionnaires was used to determine causes of disability in those studies. Therefore, it might be valuable to use less-biased community surveys and detailed clinical information to determine the status of functional disability and its causes in Japan. We examined the prevalence and underlying causes of functional disability in an elderly general population of Japanese.

## METHODS

### Study population

The Hisayama Study is a prospective cohort study of cerebrocardiovascular diseases in the town of Hisayama, a subrural community adjacent to the metropolitan area of Fukuoka, Japan.^15^ The population of the town has distributions of age, occupational status, and nutrient intake that are almost identical to those for the whole of Japan.^15^ Full community surveys of the health status and neurological conditions of residents aged 40 years or older have been repeated since 1961.^15^ One characteristic of this study is that all event data on cerebrocardiovascular diseases have been verified by detailed neurological and morphological examinations, including neuroimaging.^15^ Additionally, comprehensive surveys of functional disability and dementia in elderly adults have been carried out since 1985.^16^ Between October 2005 and August 2006, a total of 1566 residents aged 65 or older (91.5% of the total population in this age group) participated in the examination for the present study. The examination was performed in the public hall of the town or at home. In addition, we visited hospitals and health care facilities to examine institutionalized individuals. After excluding 16 subjects for whom activity of daily living (ADL) status was not available, data from 1550 subjects (601 men and 949 women) were included in the present analysis.

### Ethical considerations

This study was conducted with the approval of the Kyushu University Institutional Review Board for Clinical Research. All participants gave written informed consent, which included the purpose and procedures of the research, potential risks and benefits associated with participation, voluntary participation in the study, the right of withdrawal from the research without prejudice or penalty, and the confidentiality and security of personal data.

### Questionnaire

In the examination, each participant completed a self-administered questionnaire that inquired about sociodemographic data (including age, sex, marital status, employment status, and place of residence [domicile, hospital, long-term care facility, or nursing home]), Barthel Index items,^17^ and past history of diseases (including stroke, coronary heart disease, fracture, head injury, hypertension, diabetes, hyperlipidemia, depression, and other conditions). The completed questionnaires were reviewed by trained nurses or physicians to identify inconsistent answers and unanswered items. To diagnose dementia, all participants took neuropsychological tests (revised version of Hasegawa’s Dementia Scale [HDS-R]^18^ and Mini-Mental State Examination [MMSE]^19^), which were performed by trained nurses and physicians. Among the participants, 395 (25.2%) with test scores below the cutoff values (21/30 for the HDS-R and MMSE) underwent an additional comprehensive investigation.

### Definition of functional disability

ADL status was determined using the Barthel Index,^17^ which estimates the degree of independence in ADL of subjects by using 10 items: feeding (0, 5, or 10 points), bathing (0, 5), dressing (0, 5, 10), grooming (0, 5), bladder control (0, 5, 10), bowel control (0, 5, 10), toileting (0, 5, 10), transferring from bed to a wheelchair (0, 5, 10, 15), walking on a level surface (0, 5, 10, 15), and ascending and descending stairs (0, 5, 10). Functional disability was defined as a Barthel Index score of 95 or lower, in accordance with the definition previously reported in epidemiologic studies.^17^^,^^20^^–^^22^ In addition, the severity of disability was categorized into 3 levels as follows: slight dependence (a Barthel Index score of 95, which corresponds to 1 decrease in an item on the Barthel Index), moderate/severe dependence (a score of 25–90), and total dependence (a score of 0–20, which corresponds approximately to a bedridden state, with at least 8 decreased items).^17^

### Cause of disability

To determine the cause of functional disability, all available past clinical information, including medical records and findings from neurologic examination and brain imaging studies, which was gathered by using the follow-up system of the Hisayama Study,^15^^,^^23^ was reviewed independently by 2 of the authors (D.Y. and T.N.). Any disagreement in cause attribution was resolved by a consensus of a panel of the authors (D.Y., T.N., and Y.K). If a subject had 2 or more conditions that impaired ADL, the disease that contributed to the deterioration of at least 1 category of ADL level (eg, from moderate/severe dependence to total dependence) was defined as the major cause. For instance, if a subject had mild gait disturbance caused by stroke but gradually became bedridden due to subsequent dementia, dementia would be considered the major cause, whereas stroke would be selected if the subject became bedridden soon after a severe stroke event, even if the participant later developed dementia. Among the 311 disability cases, the 2 researchers completely agreed on the cause of functional disability in 242 (77.8%) cases. In the remaining 68 (22.1%) cases, a consensus on the cause was reached after discussion.

Causes of disability were categorized into 4 groups: dementia (vascular dementia, Alzheimer disease, and other dementia), stroke (ischemic stroke and hemorrhagic stroke), orthopedic disease (fracture, arthritis, rheumatoid arthritis, and other orthopedic disease), and other disease. Dementia and its subtypes were diagnosed according to the guidelines of the Diagnostic and Statistical Manual of Mental Disorders, Third Edition, Revised (DSM-III-R),^24^ the criteria of the National Institute of Neurological and Communicative Disorders and Stroke–Alzheimer’s Disease and Related Disorders Association,^25^ and the criteria of the National Institute of Neurological Disorders and Stroke–Association International pour la Recherche et l’Enseignement en Neurosciences.^26^ Stroke was defined as the sudden onset of nonconvulsive and focal neurologic deficits persisting at least 24 hours. A diagnosis of stroke and its subtypes was determined on the basis of medical records and brain imaging studies.^27^ Hemorrhagic stroke included brain hemorrhage and subarachnoid hemorrhage. The diagnosis and classification of orthopedic disease were determined with clinical information available from the questionnaire, medical records, and annual health examinations.

### Statistical analysis

The software package SAS (version 9.2; SAS Institute, Cary, NC, USA) was used to perform all statistical analyses. The Student *t*-test was used to compare continuous variables, and the chi-square test was used to evaluate proportions. We calculated the prevalences of disability with 95% confidence intervals (CIs) by using a binary distribution. Trends in the prevalence of disability across 5-year age categories were tested by means of logistic regression analysis. A 2-sided *P* value less than 0.05 was considered statistically significant in all analyses.

## RESULTS

The characteristics of study subjects according to functional disability status are shown in Table 1. The mean overall age was 76 years, and the proportion of women was 61.1%. A total of 311 subjects (85 men and 226 women) had some type of functional disability, resulting in a prevalence of 20.1%. As compared with those without disability, subjects with disability were more likely to be older, female, unemployed, living alone, and institutionalized. Among those with disability, the proportions of subjects with slight, moderate/severe, and total dependence were 25.4%, 49.8%, and 24.8%, respectively.

**Table 1.** Characteristics of study population by functional disability (Hisayama Study, 2005)

	All subjects (*n* = 1550)	Subjects without disability (*n* = 1239)	Subjects with disability (*n* = 311)	*P*-value^a^
Age, mean ± SD	75.8 ± 7.3	74.2 ± 6.3	82.1 ± 7.7	<0.001
Women, %	61.1	58.2	72.7	<0.001
Current working status, %				<0.001
Unemployed/retired/housewife	73.1	68.9	90.4	
Working	26.9	31.1	9.6	
Marital status, %				<0.001
Never married	2.5	2.2	3.5	
Married	63.4	68.6	42.8	
Divorced/widowed/separated	34.1	29.2	53.7	
Living arrangement, %				0.04
Living alone	10.9	10.1	14.2	
Living with others	89.1	89.9	85.8	
Place of residence, %				<0.001
Home	91.6	99.3	60.5	
Hospital	5.2	0.6	23.8	
Health care facility	3.2	0.1	15.7	
ADL disability level, %				
Slight dependence	5.0	—	25.4	
Moderate/severe dependence	10.0	—	49.8	
Total dependence	5.1	—	24.8	

^a^*P* value, comparison between subjects with and without disability.

As shown in Table 2, the prevalence of functional disability increased with age, with a doubling in prevalence for every 5-year increment. The prevalence of disability was significantly higher in women than in men (*P* < 0.001), especially among participants aged 85 or older (*P* = 0.01). A comparable relationship was observed in subjects with total dependence, whereas the prevalence of slight and moderate/severe dependence was not significantly different between sexes in any age category (data not shown).

**Table 2.** Prevalence of disability by age category (Hisayama Study, 2005)

Age category	Total (*n* = 1550)		Men (*n* = 603)		Women (*n* = 947)		*P* value between sexes
		
	No. with disability/ participants	Prevalence, % (95% CI)	No. with disability/ participants	Prevalence, % (95% CI)	No. with disability/ participants	Prevalence, % (95% CI)	
65–69	18/366	4.9 (2.9–7.7)	9/161	5.6 (2.6–10.4)	9/205	4.4 (2.0–8.2)	0.60
70–74	38/393	9.7 (6.9–13.0)	14/171	8.2 (4.6–13.4)	24/222	10.8 (7.1–15.7)	0.38
75–79	53/331	16.0 (12.2–20.4)	18/129	14.0 (8.5–21.2)	35/202	17.3 (12.4–23.3)	0.41
80–84	75/256	29.3 (23.8–35.3)	20/91	22.0 (14.0–31.9)	55/165	33.3 (26.2–41.1)	0.06
85+	127/204	62.3 (55.2–68.9)	24/51	47.1 (32.9–61.5)	103/153	67.3 (59.3–74.7)	0.01
All ages	311/1550	20.1 (18.1–22.2)	85/603	14.1 (11.4–17.1)	226/947	23.9 (21.1–26.7)	<0.001
*P* for trend		<0.001		<0.001		<0.001	

**Figure 1.**
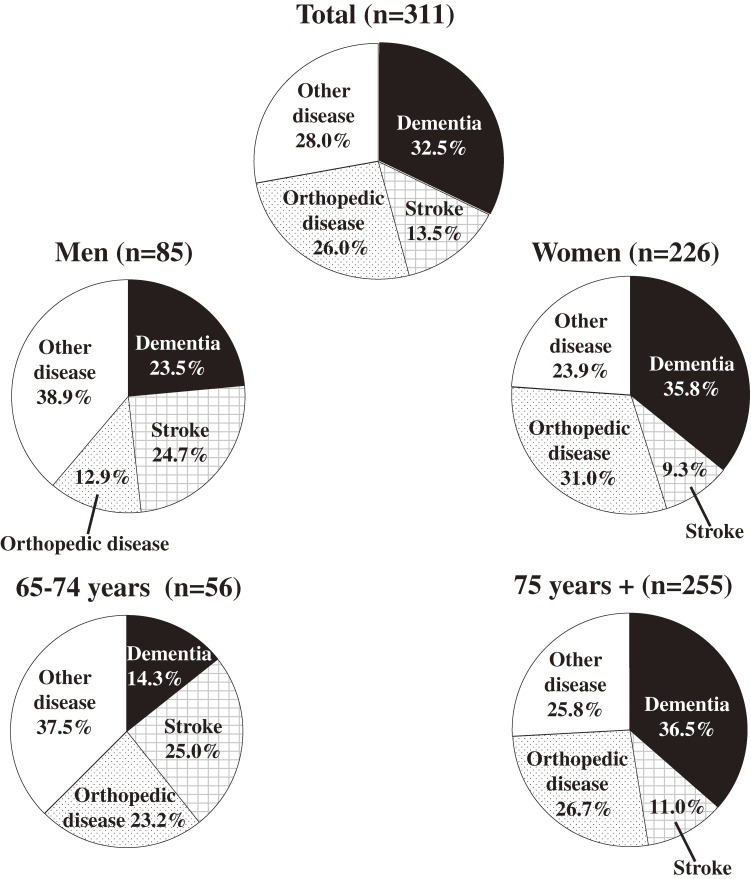
Causes of functional disability by sex and age (Hisayama Study, 2005).

Next, we investigated the causes of functional disability (Figure 1). Among the 311 disability cases, dementia accounted for 32.5%, stroke for 13.5%, orthopedic disease for 26.0%, and other disease for 28.0% of cases. Among the 101 subjects with dementia-related disability, 22 (21.8%) had a history of a stroke events that resulted in slight or moderate/severe dependence. When the results were categorized by sex, dementia accounted for 23.5%, stroke for 24.7%, orthopedic disease for 12.9%, and other disease for 38.9% of cases of functional disability in the 85 disabled men; the respective values were 35.8%, 9.3%, 31.0%, and 23.9% in the 226 disabled women. Stroke was the most common cause of disability in men, whereas dementia and orthopedic disease were more frequent in women. When the findings were analyzed by age category, dementia accounted for 14.3%, stroke for 25.0%, orthopedic disease for 23.2%, and other disease for 37.5% of disability cases in subjects aged 65 to 74 years; the respective proportions were 36.5%, 11.0%, 26.7%, and 25.8% for subjects aged 75 or older; that is, dementia was the most frequent cause of disability in subjects aged 75 or older, whereas stroke was the most common cause in subjects aged 65 to 74 years.

The subtypes of causes of functional disability by sex are shown in Table 3. Among cases of dementia, vascular dementia was most frequent in men (12.9%), whereas Alzheimer disease was most common in women (15.0%). With regard to stroke subtype, ischemic stroke was more frequent in men than in women (17.6% vs 6.2%). With regard to orthopedic disease, the proportions of fracture and arthritis were higher, especially in women (15.0% and 10.2%, respectively).

**Table 3.** Subtypes of causes of disability by sex (Hisayama Study, 2005)

Disease/condition	Total (*n* = 311)		Men (*n* = 85)		Women (*n* = 226)		*P*-value^a^
		
	Number	%	Number	%	Number	%	
Dementia	101	32.5	20	23.5	81	35.8	0.04
Vascular dementia	30	9.6	11	12.9	19	8.4	0.23
Alzheimer disease	40	12.9	6	7.1	34	15.0	0.06
Other dementia	31	10.0	3	3.5	28	12.4	0.02
Stroke	42	13.5	21	24.7	21	9.3	<0.001
Ischemic stroke	29	9.3	15	17.6	14	6.2	0.002
Hemorrhagic stroke	13	4.2	6	7.1	7	3.1	0.20
Orthopedic disease	81	26.0	11	12.9	70	31.0	0.001
Fracture	38	12.2	4	4.7	34	15.0	0.01
Arthritis	25	8.0	2	2.4	23	10.2	0.03
Rheumatoid arthritis	11	3.5	2	2.4	9	4.0	0.73
Other orthopedic disease	7	2.3	3	3.5	4	1.8	0.40
Other disease	87	28.0	33	38.8	54	23.9	0.009

^a^*P* value for comparison between sexes.

**Figure 2.**
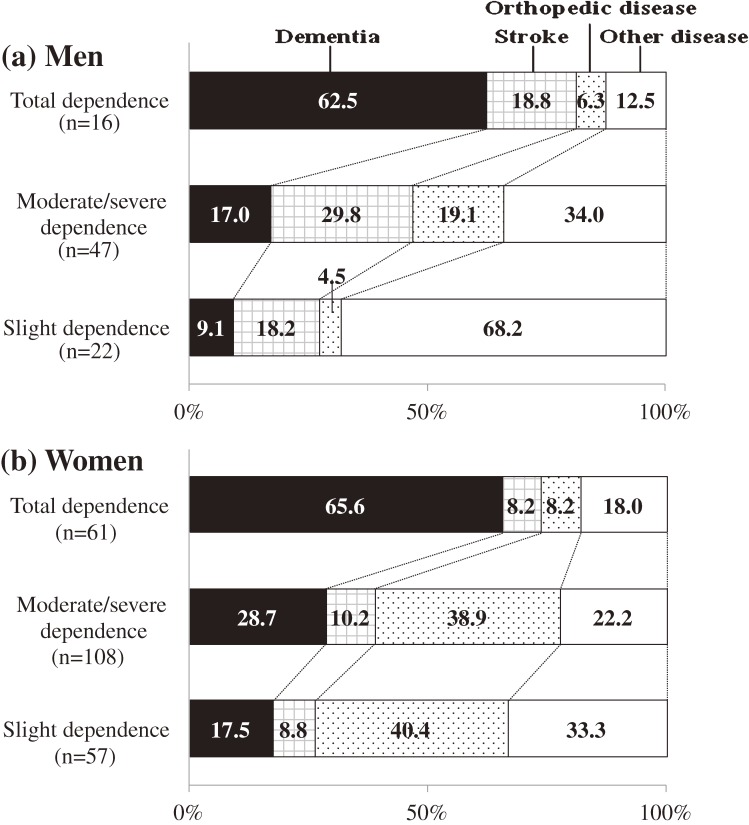
Causes of functional disability by severity of disability in men and women (Hisayama Study, 2005). Total dependence: Berthel Index score = 0–20. Moderate/severe dependence: Berthel Index score = 25–90. Slight dependence: Berthel Index score = 95.

Figure 2 shows the causes of functional disability among the 311 subjects according to disability severity by sex. In subjects with total dependence, dementia was the most frequent cause in both sexes: the proportion was 62.5% in men and 65.6% in women. In subjects with slight or moderate/severe dependence, stroke was the most common cause of disability in men, whereas orthopedic disease was the most frequent in women.

Finally, we investigated place of residence in the 311 disabled subjects according to functional severity. Among subjects with slight dependence, 91.1% lived at home, 6.3% were hospitalized, and 2.6% stayed in health care facilities; the respective values were 72.3%, 17.4%, and 10.3% for those with moderate/severe dependence. In contrast, among subjects with total dependence, only 5.2% lived at home, whereas 54.6% and 40.2% stayed in a hospital or health care facility, respectively.

## DISCUSSION

The present study demonstrated that the prevalence of functional disability was 20.1% in an elderly general population of Japanese. Additionally, we found that the prevalence of disability increased steeply with age, with a doubling of prevalence for each 5-year increment. Prevalence was higher in women than in men, especially in individuals aged 85 or older. Importantly, in our subjects the major cause of disability was stroke in men and dementia in women. In particular, dementia was the most common cause of disability in subjects with total dependence, most of whom required full-time care in hospitals or health care facilities. These findings highlight the clinical importance of effective strategies for preventing dementia. Such strategies could reduce the social and economic burden of functional disability among elderly Japanese.

### Prevalence of disability

There is considerable divergence in the prevalence of disability reported in community-based studies, with values ranging from 6% to 34.5%.^4^^–^^13^ For aged Japanese populations, these studies have reported a disability prevalence ranging from 8% to 17%,^10^^–^^13^ which is lower than that obtained in the present study. A possible reason for this discrepancy is the difference in the proportion of old old adults in the studies, as this group is at high risk for functional disability. Among people aged 65 years or older, the proportion of those aged 85 years or older was 4.5% to 8.7% in previous studies, which were conducted from 1977 to 1996,^1^^,^^10^^–^^13^ as compared with 11.4% in the present study, performed in 2005. These findings indicate that the proportion of old old has increased over time in Japan, which has led to a recent increase in the prevalence of functional disability. In addition, some selection bias was likely in previous studies, because subjects staying in hospitals or health care facilities might not have been fully examined. In contrast, the participation rate was high (91%) in our study, and we included institutionalized subjects in the study to minimize selection bias. This bias in previous studies would lead to underestimation of the prevalence of disability. Furthermore, the discrepant findings may have been due to a difference in the definition of disability across studies. The Barthel Index, which was used in our study, has been reported to be more sensitive in detecting disability as compared with other indices with fewer ADL domains (eg, the Katz Index), which were used in other studies.^6^^,^^28^ Indeed, in a sensitivity analysis using the Katz Index—in which functional disability was defined as need for assistance in 1 or more activities of 6 ADL domains, including feeding, bathing, dressing, toileting, transferring, and continence—the prevalence of disability declined to 18.3% in our study.

### Sex differences in disability

In our study, the prevalence of disability was higher in women than in men, especially among persons aged 85 or older. Comparable findings were observed in previous community-based studies in Sweden and Japan.^8^^,^^29^^,^^30^ However, there is no consensus on the interpretation of this sex difference. A possible explanation is that there are sex differences in death rates for underlying diseases; that is, women might survive with some form of disability after developing cardiovascular disease, whereas men might be more likely to die immediately after the incident disease, since the underlying comorbidity may be more severe in men than in women.^31^^,^^32^ Another possible explanation is that musculoskeletal disease may have a greater influence on functional limitations in women than in men. For example, a population-based study in the United States indicated that musculoskeletal impairments were attributed to disability more frequently in women than in men.^33^ In our subjects, disabled women also had a greater incidence than men of orthopedic diseases such as fracture and arthritis.

### Cause of disability

In the present study, dementia was the most frequent cause of functional disability in both sexes, especially among those aged 75 or older. In agreement with this finding, the Adult Health Study in Hiroshima, Japan and a community-based study in Stockholm, Sweden showed that dementia had a greater influence on the development of disability and ADL decline than did stroke, orthopedic disease, or other chronic diseases.^34^^,^^35^ Furthermore, our study found that the proportion of stroke was high in subjects aged 65 to 74 years. Previous community-based prospective studies in Japan and the United States have also shown that stroke was associated with risk of functional disability.^36^^–^^38^ A systematic review reported that more than one-third of patients with recurrent stroke later developed dementia.^39^ We also revealed that 21.8% of subjects with dementia-related disability had a history of stroke events with slight or moderate/severe dependence. These findings indicate that it is important to prevent stroke events to reduce the risk of future dementia and total dependence. Interestingly, orthopedic disease such as fracture and arthritis contributed mainly to slight dependence and moderate/severe dependence in women. Further investigations will be needed to determine the effect of orthopedic disease on subsequent ADL level.

### Place of residence and severity of disability

To date, few studies of general populations have classified ADL level according to place of residence. In our study, approximately 95% of subjects with total dependence were institutionalized in hospitals or health care facilities. Most of these subjects had dementia and were bedridden. The increase in patients hospitalized or staying in health care facilities is a major social and economic burden in Japan. Therefore, it is imperative to establish effective strategies for preventing the development of dementia and subsequent deterioration of ADL.

### Study strengths and limitations

The strength of our study is that selection bias was minimized by including more than 90% of all Hisayama residents aged 65 years or older and by examining subjects staying in hospitals and health care facilities. In addition, cardiovascular events and dementia were evaluated using not only questionnaires but also detailed clinical information, as these parameters are main endpoints of the ongoing Hisayama Study.^15^^,^^23^ A limitation is that this was a cross-sectional study. Consequently, causal relationships cannot be inferred between underlying diseases and functional disability.

### Conclusion

Our study revealed that functional disability is common among Japanese elderly adults and that dementia is the most frequent cause of disability, especially in persons with total dependence. Stroke is a major cause of disability in men and in individuals aged 65 to 74 years (the young old). In countries such as Japan, where the elderly population is increasing rapidly, it is important to establish effective prevention strategies for dementia and stroke to reduce the risk of disability and extend healthy life expectancy in later life.
